# TreeSeg-Net: An End-to-End Instance Segmentation Network for Leaf-Off Forest Point Clouds Using Global Context and Spatial Proximity

**DOI:** 10.3390/plants15040525

**Published:** 2026-02-07

**Authors:** Xingmei Xu, Ruihang Zhang, Shunfu Xiao, Jiayuan Li, Xinyue Zhang, Liying Cao, Helong Yu, Yuntao Ma, Jian Zhang, Xiyang Zhao

**Affiliations:** 1 College of Information Technology, Jilin Agricultural University, Changchun 130118, Chinaruihang.zhang@mails.jlau.edu.cn (R.Z.); yuhelong@jlau.edu.cn (H.Y.);; 2 College of Land Science and Technology, China Agricultural University, Beijing 100193, China; 3 Faculty of Agronomy, Jilin Agricultural University, Changchun 130118, China; 4 Department of Biology, University of Columbia Okanagan, Kelowna, BC V1V 1V7, Canada; 5 College of Forestry and Grassland Science, Jilin Agricultural University, Changchun 130118, China

**Keywords:** precision forestry, point cloud segmentation, TreeSeg-Net, UAV photogrammetry, remote sensing

## Abstract

Forest ecosystems play a pivotal role in maintaining the balance of the global carbon cycle and conserving biodiversity. High-density point clouds derived from unmanned aerial vehicle (UAV) structure from motion (SfM) and multi-view stereo (MVS) technologies offer a cost-effective solution for data acquisition. These technologies have become efficient tools for facilitating precision forest resource management and extracting individual tree structural parameters. However, in complex forest scenarios during the leaf-off season, canopies exhibit unstructured branch network morphologies due to the absence of leaf occlusion, and adjacent crowns are heavily interlaced. Consequently, existing segmentation methods struggle to overcome challenges associated with fuzzy boundaries and instance adhesion. To address these challenges, this study proposes TreeSeg-Net, an end-to-end instance segmentation network designed to precisely separate individual trees directly from raw point clouds. The network incorporates a global context attention module (GCAM) to capture long-range feature dependencies, thereby compensating for the limitations of sparse convolution in perceiving global information. Simultaneously, a spatial proximity weighting module (SPWM) is designed. By introducing geometric center constraints and a distance penalty mechanism, this module effectively mitigates under-segmentation issues caused by the feature similarity of adjacent branches in high-canopy-density environments. Experimental results demonstrate that TreeSeg-Net achieves an average precision (AP) of 97.2% in instance segmentation tasks and a mean intersection over union (mIoU) of 99.7% in semantic segmentation tasks. Compared to mainstream networks, the proposed method exhibits superior segmentation accuracy, providing an efficient and automated technical solution for precise resource inventory in complex forest environments.

## 1. Introduction

Forest ecosystems, as core components of the Earth’s biosphere, play an indispensable role in maintaining ecological balance, mitigating climate change, conserving biodiversity, and ensuring sustainable forest management [1,2,3]. To achieve precision forest resource management and ecological assessment, the extraction of structural parameters at the individual tree scale is a central task in forest inventory [4]. Within this context, high-precision individual tree segmentation (ITS) serves as a critical step, providing foundational data support for extracting key phenotypic parameters such as tree height, diameter at breast height (DBH), and biomass [5,6]. UAV-based SfM and MVS technologies, offering a cost-effective solution, enable the reconstruction of high-density forest point clouds through overlapping imagery [7,8]. However, in complex forest scenarios, stably and accurately isolating individual tree instances from these point clouds remains one of the primary challenges in current 3D forest analysis [9,10].

Addressing the ITS task, early works primarily relied on canopy height models (CHMs) and 2D image processing techniques, utilizing algorithms such as local maxima detection [11], watershed segmentation [12], and graph cuts [13] to identify and delineate individual tree crowns [14]. While these methods exhibit high efficiency in regular stands, their segmentation accuracy is often limited in areas with homogeneous tree heights or severe canopy interlacing, as 2D projection inevitably compromises detailed 3D structural information [15,16,17]. To mitigate information loss, an increasing number of studies have shifted towards performing segmentation directly on 3D point clouds, identifying individual trees through clustering, region growing, or geometric feature-based strategies [18,19,20]. Nevertheless, in real-world forest scenarios characterized by significant morphological variations and mutual canopy occlusion, these methods—reliant on explicit rules and geometric assumptions—still struggle to maintain robust performance across diverse stand conditions.

In recent years, driven by the rapid advancements of deep learning in computer vision, researchers have increasingly applied these techniques to 3D point cloud segmentation tasks [21,22,23]. Distinct from traditional rule-based or geometric feature-dependent methods, deep neural networks can automatically learn complex shape and texture features directly from raw point clouds, thereby exhibiting superior adaptability to forest environments characterized by diverse canopy morphologies and severe occlusion [24]. Deep learning-based approaches for ITS generally fall into two categories: proposal-based frameworks and proposal-free approaches. Proposal-based methods typically follow a detect-then-segment paradigm, locating potential tree regions before predicting instance masks. By explicitly modeling potential instance regions, these methods achieve precise object localization and boundary cropping in complex scenes. Representative works include SGPN [25], 3D-MPA [26], 3D-SIS [27], and PointRCNN [28], which is based on two-stage detectors. Conversely, proposal-free methods circumvent the candidate generation process. They usually rely on point-level semantic prediction combined with clustering or center regression in a feature embedding space to directly recover instance structures. These approaches offer advantages in computational efficiency and scalability, as seen in PointGroup [29], SoftGroup [30], and CPSeg [31].

Furthermore, several studies have incorporated forestry priors or geometric constraints, integrating deep learning with physically meaningful grouping strategies. For instance, Xia et al. [32] combined RandLA-Net with MeanShift to extract individual trees from point clouds. Henrich et al. [33], in their TreeLearn framework, achieved instance separation in an offset space by predicting offset vectors pointing to the trunk base. Addressing mobile laser scanning (MLS) data, Jiang et al. [34] developed a segmentation strategy combining cylindrical convolution with dynamic shifting to resolve segmentation challenges caused by canopy overlap and occlusion in complex urban scenarios. Additionally, while Sun et al. [35] proposed a sparse 3D U-Net, Huo et al. [3] improved upon it by introducing multi-head attention mechanisms. By enhancing global and multi-scale feature capture through subspace projection, this method effectively achieved ITS on smartphone-acquired point clouds. Collectively, these studies demonstrate that combining point-level predictions based on deep features with spatial clustering holds significant promise for processing irregular and structurally complex forest point clouds.

Despite the significant progress of existing deep learning methods in point cloud processing, two core challenges persist. First, the inherent locality of sparse convolution limits the network’s ability to comprehend the global structure of an entire tree. Second, clustering or mask generation mechanisms based solely on feature similarity fail to effectively distinguish adjacent branches that are spatially entangled but indistinguishable in feature space. To address these issues, this study posits two research hypotheses: (1) incorporating long-range dependency modeling can compensate for the limited receptive field of sparse convolution, and (2) introducing explicit geometric constraints can effectively differentiate spatially entangled instances. Driven by these hypotheses, the primary aim of this study is to develop TreeSeg-Net, an end-to-end instance segmentation framework integrating global perception and geometric constraints. Obviating the need for complex post-processing steps or manual intervention, the model takes raw forest point clouds as input and automatically outputs ITS results. Comprehensive experiments were conducted on SfM point cloud datasets generated by consumer-grade UAVs to validate the method’s effectiveness under complex stand conditions. To achieve this objective, the specific contributions are summarized as follows:

1. A novel end-to-end instance segmentation network, TreeSeg-Net, is proposed for complex forest scenarios. The network integrates an improved sparse 3D U-Net with a transformer decoder.
2. A GCAM and a SPWM are designed. The GCAM is designed to capture long-range feature dependencies, compensating for the limitations of sparse convolution in global information perception. The SPWM introduces geometric center constraints and a distance penalty mechanism to address the challenges of boundary fuzziness and instance adhesion caused by feature similarity among adjacent canopies in high-density environments.
3. Through comparative analysis with various mainstream point cloud segmentation networks and ablation studies, the proposed method proves to be highly effective, providing an efficient and economical technical solution for forest resource inventory.

## 2. Materials and Methods

### 2.1. Study Area and Data Acquisition

**Figure 1:**
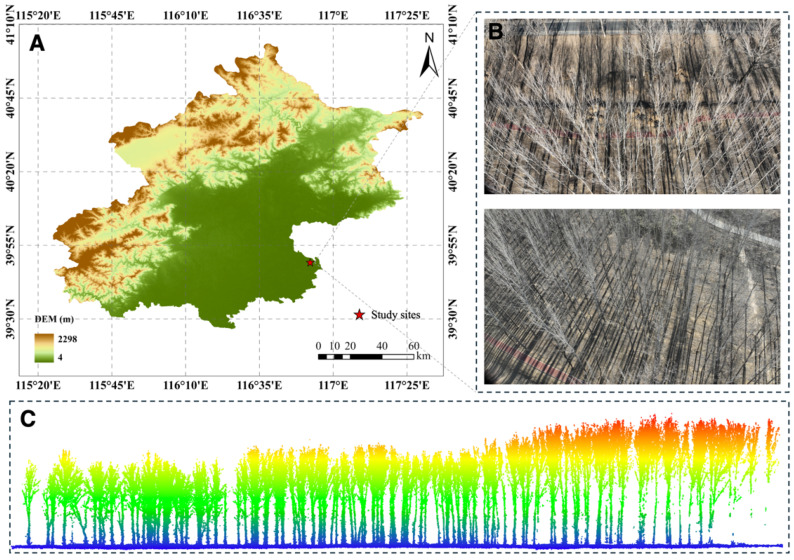
Overview of the study area: (**A**) Schematic diagram of the geographical location. (**B**) Field photograph showing the environment above the canopy within the sample plot. (**C**) Side view of the forest point cloud.

The experiment was conducted in October 2024 in Tongzhou District, Beijing, China (39.82° N, 116.87° E). The geographical location of the study site is illustrated in Figure 1A. The experimental area covers a total extent of approximately 4.36 hectares and features flat and open terrain with an average elevation of approximately 20 m. The region belongs to a warm temperate continental semi-humid monsoon climate zone, characterized by distinct seasons, an annual average temperature of 12.7 °C, and annual average precipitation of 445.6 mm. The stand type is an artificial plantation, with *Populus tomentosa* Carr. as the dominant species. As shown in Figure 1B, the trees are arranged relatively neatly. Since data acquisition occurred during the early leaf-off season, the canopy lost its leaf occlusion, presenting a complex, unstructured branch network morphology (Figure 1B). Figure 1C provides a side view of the forest point cloud, showcasing the vertical structure of the stand. Additionally, the acquired point cloud exhibits a high average density of 1339 points/m^2^, providing rich geometric details for individual tree segmentation.

**Figure 2:**
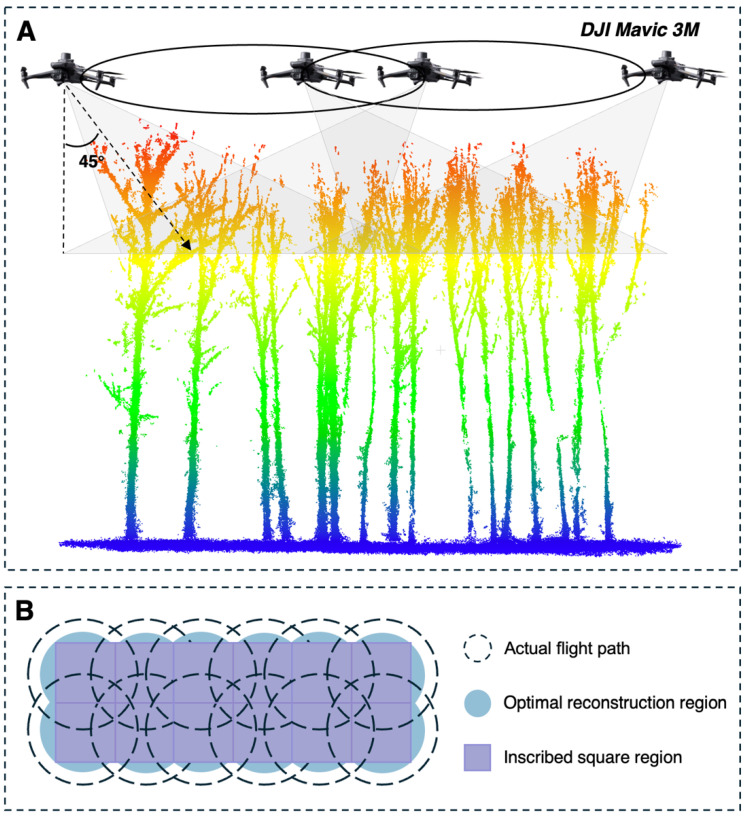
(**A**) Schematic diagram of the data acquisition strategy. (**B**) Bird’s-eye view of the CCO flight path, with an overlap rate of 50% between circles.

To acquire high-quality forest point clouds containing fine trunk textures and complete branching structures, a DJI Mavic 3M (DJI, Shenzhen, China) UAV platform was employed for image data collection. A cross-circle oblique (CCO) flight path (Figure 2) was adopted to capture canopy images [36]. The flight altitude of the CCO path was set to 47 m above the canopy top, with a flight speed maintained at 5 m/s. The gimbal pitch angle was fixed at 45°. This flight plan consisted of multiple intersecting circular paths, with 35 waypoints set for each circle and an overlap rate of 50% between adjacent circles, resulting in a total of 537 multi-view raw images. Simultaneously, a standard nadir flight path was used to obtain orthophotos of the canopy. The nadir flight altitude was set to 80 m, with the speed also maintained at 5 m/s. The forward and side overlap rates were set to 80% and 70%, respectively, yielding 180 images. All flight missions were executed under meteorological conditions with uniform lighting and low wind speeds to ensure image clarity.

### 2.2. Data Preprocessing

Mosaicking of raw images and generation of 3D point clouds were performed using Agisoft Metashape Professional (Agisoft LLC, St. Petersburg, Russia, version 2.1.0). The software operates based on the standard SfM-MVS pipeline: first, the SfM algorithm estimates internal and external camera parameters to construct a sparse point cloud; subsequently, the MVS algorithm further refines the model to generate a dense 3D representation. To ensure point cloud density and geometric accuracy, parameters for both photo alignment and dense cloud generation were set to “High Quality”.

**Figure 3:**
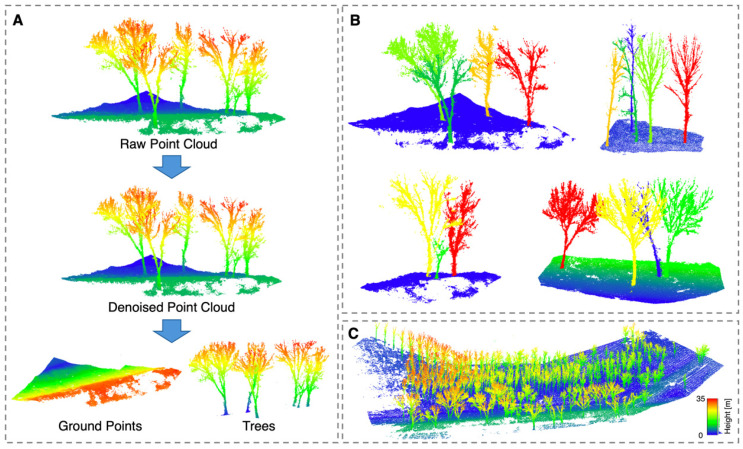
Schematic diagram of the data preprocessing workflow: (**A**) Filtering process: raw point clouds are denoised via the SOR algorithm, followed by ground point removal using the CSF algorithm to extract vegetation points. (**B**) Dataset samples for training, where each sample contains several trees and surrounding ground points. (**C**) The complete plot point cloud.

To construct a standardized dataset suitable for TreeSeg-Net, the raw point clouds underwent a series of normalization procedures. First, the statistical outlier removal (SOR) algorithm was employed to eliminate discrete noise generated during the reconstruction process. Subsequently, the cloth simulation filter (CSF) algorithm was utilized to classify the point cloud into ground and vegetation categories (Figure 3A). To address the inherent class imbalance where ground points significantly outnumbered tree points, a random downsampling strategy was applied to the ground category to achieve a balanced distribution. Afterwards, height normalization was performed on the processed point cloud based on the extracted digital elevation model (DEM) to mitigate the interference of terrain undulation on tree height features. Finally, given the massive data volume of the entire plot, the normalized point cloud was partitioned into blocks of 10 m by 10 m using a sliding window strategy with overlap (Figure 3B). This approach was adopted to mitigate boundary effects and increase data diversity, resulting in a total of 146 blocks, where each block contained approximately four to five trees. The larger sub-region, consisting of 111 blocks, was randomly split into training and validation sets at an approximate ratio of 8 to 2. Meanwhile, the smaller sub-region of 35 blocks served exclusively as the independent test set to evaluate model generalization.

### 2.3. Overall Architecture

**Figure 4:**
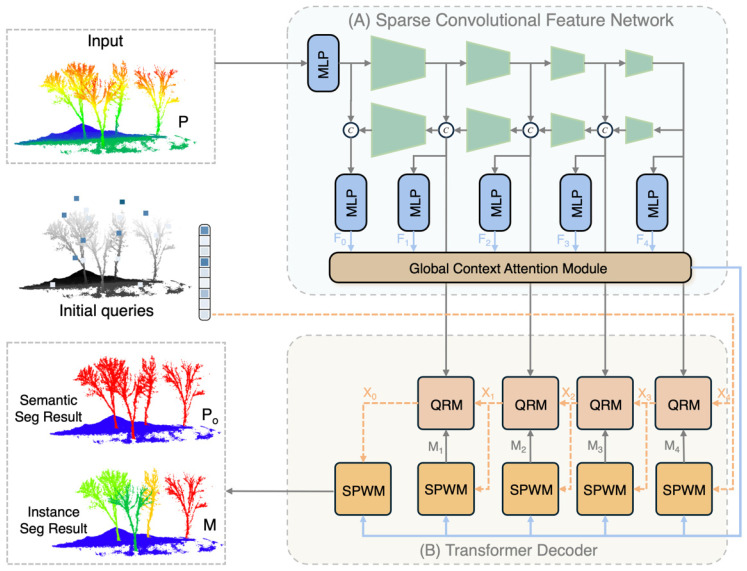
Overall architecture of TreeSeg-Net: (**A**) Sparse convolutional feature network. (**B**) Transformer decoder.

In leaf-off forest scenarios, severely interlaced branches and highly similar geometric features present significant segmentation challenges. Addressing these, existing clustering-based methods often struggle to delineate clear boundaries within complex skeletal structures. Therefore, drawing inspiration from Organ3DNet [37], this study designed TreeSeg-Net. The network follows the mask prediction paradigm, discarding the indirect approach of mapping point clouds to a feature space for clustering. Instead, it directly learns and predicts binary masks representing individual tree instances in an end-to-end manner. As shown in Figure 4, the main architecture of TreeSeg-Net comprises two core modules: the sparse convolutional feature network and the transformer decoder. The network takes raw point cloud coordinates *P* as input and, through feature encoding and decoding interactions, synchronously outputs point-level semantic classes *P_o_* and instance masks *M*.

The sparse convolutional feature network primarily functions as the encoder. Addressing the characteristics of extremely high sparsity and non-uniform distribution inherent in forest point cloud data, this module adopts a sparse convolutional backbone based on the U-Net structure. As shown in Figure 4A, this network extracts multi-scale features *F*_0_ to *F*_4_, ranging from local geometric textures to high-level semantics, through multi-level downsampling and upsampling operations. However, standard sparse convolution focuses mainly on local neighborhood information, making it difficult to capture the macrostructure of large-scale forest plots. To resolve this limitation, TreeSeg-Net introduces a GCAM at the end of the backbone network. The GCAM establishes long-range feature dependencies and fuses global context information into local features, thereby enhancing the encoder’s feature representation capabilities in environments with complex occlusion.

The Transformer decoder is responsible for parsing the deep features extracted by the encoder into specific individual tree instances. This module first initializes a set of learnable instance queries, which are then progressively optimized through a multi-level cascading approach. As illustrated in Figure 4B, the decoding process alternates between a QRM and a SPWM. The QRM utilizes self-attention and cross-attention mechanisms to facilitate information interaction among queries and between queries and pixel-level features. To address the insufficient distinctiveness of original mask modules when processing overlapping canopies, this study designed the SPWM. Unlike traditional methods that rely solely on feature similarity, the SPWM introduces explicit geometric spatial constraints during the decoding phase. This forces the network to balance feature consistency with spatial proximity, effectively resolving the issue of branch entanglement between adjacent trees.

### 2.4. Sparse Convolutional Feature Network

Point cloud data in forest scenarios are typically massive, unordered, and highly sparse in 3D space [38]. Directly employing traditional voxelized 3D convolutional networks would result in a significant waste of computational resources by processing many empty voxels. Furthermore, such an approach would fail to meet the real-time requirements of high-throughput phenotypic analysis. To balance computational efficiency with feature extraction accuracy, this study adopts a sparse 3D convolutional network built on the Minkowski Engine as the backbone architecture. This backbone follows the classic U-Net design paradigm, achieving deep abstraction and fusion of multi-scale features through an encoder–decoder structure.

Before entering the network, the raw point cloud coordinates *P* are voxelized and quantized into a sparse tensor. The encoder stage consists of a series of stacked sparse convolutional layers and pooling operations. As the network depth increases, the spatial resolution of the feature maps gradually decreases, while the channel dimension increases, transforming low-level geometric textures into abstract high-level semantic features. The decoder stage progressively restores spatial resolution via transposed convolution and fuses high-resolution features from the corresponding encoder levels into the decoding path through skip connections. This design effectively mitigates the loss of spatial information during downsampling, enabling the network to output hierarchical features at five scales, from *F*_0_ to *F*_4_ (Figure 4A). These multi-scale features not only preserve the details necessary for describing trunks and branches but also contain semantic information to distinguish different vegetation levels.

#### Global Context Attention Module (GCAM)

**Figure 5:**
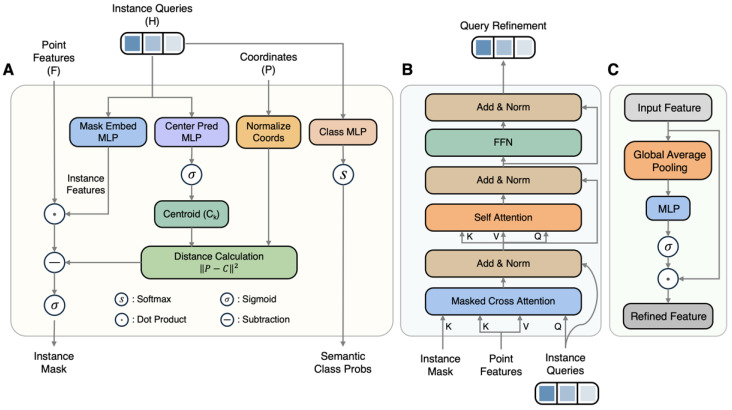
Structural diagram of the core modules in TreeSeg-Net: (**A**) SPWM introduces a geometric center prediction branch to impose spatial distance penalties. (**B**) QRM utilizes mask cross-attention mechanisms to aggregate features. (**C**) GCAM performs feature channel recalibration via global average pooling and an MLP.

Although sparse convolutional networks perform well in processing large-scale point clouds, the receptive field of their convolution kernels is inherently restricted to local neighborhoods. In forest plots with high canopy density, individual tree crowns often occupy large spatial ranges, and there is severe branch interlacing between adjacent trees. Relying solely on local features makes it difficult for the network to perceive the structure of the entire tree at a macroscopic level. This often leads to the erroneous fragmentation of a single tree into multiple instances or the confusion of adhered parts of adjacent trees. To overcome this limitation, TreeSeg-Net introduces a GCAM at the neck of the backbone network. The core objective of the GCAM is to establish long-range feature dependencies, enabling the network to perceive the global distribution patterns of the entire plot before aggregating local features. As shown in Figure 5C, the GCAM is a lightweight and efficient attention branch.

Specifically, for a given feature tensor $F_{i}\in R^{N\times C}$, where *N* is the number of voxels and *C* is the number of channels, the module first compresses the information from the spatial dimension into a channel descriptor $z\in R^{1\times C}$ via global average pooling. For the *c*-th channel, its global statistic *z_c_* is calculated as follows:

(1) $$z_{c}=\frac{1}{N}\sum_{i=1}^{N}F_{in}^{c}(i)$$

where $F_{in}^{c}(i)$ represents the response value of the input feature at the *c*-th channel and the *i*-th voxel. Subsequently, to capture non-linear interactions between channels, a gating mechanism is employed to adaptively generate a channel weight vector $s\in R^{C}$ and recalibrate the original features:

(2) $$F_{out}=\sigma \left(W_{2}\delta \left(W_{1}z\right)\right)\times F_{in}$$

where δ denotes the ReLU activation function, σ denotes the Sigmoid activation function, and *W*_1_ and *W*_2_ are the learnable weight parameters of the MLP. Through this mechanism, the GCAM explicitly models the correlations between channels. It adaptively enhances those feature channels that possess critical discriminative power for distinguishing individual tree instances—such as the texture features at the junction of trunks and crowns—while simultaneously suppressing environmental noise.

### 2.5. Transformer Decoder

Following the extraction and enhancement of global context features by the sparse convolutional backbone, the Transformer decoder parses these high-dimensional features into specific individual tree instances. As illustrated in Figure 4B, the decoder of TreeSeg-Net adopts a mask classification paradigm based on set prediction. Unlike traditional pipelines that perform point-wise classification followed by clustering, this module utilizes a set of learnable instance queries to represent potential tree objects. During inference, these queries function as soft anchors to probe individual tree features within the scene. The decoder is composed of multiple cascaded decoding layers, employing an iterative optimization strategy to progressively refine segmentation accuracy. In each layer, instance queries first enter the QRM, where they aggregate multi-scale features and contextual information via attention mechanisms to optimize their feature representation. Subsequently, the updated queries are fed into the SPWM to generate instance mask predictions and classification probabilities for the current level. This design enables the network to infer both the category semantics and geometric shapes of all trees in the scene in parallel. Operating within an end-to-end framework, it eliminates the need for complex post-processing steps.

#### 2.5.1. Query Refinement Module (QRM)

The QRM leverages attention mechanisms to aggregate context information extracted by the backbone into instance queries, thereby updating their feature representations (Figure 5B). This module accepts initialized queries or output vectors from the preceding layer as input and progressively optimizes the query state through three internal sub-layers. First, a self-attention mechanism establishes communication channels among instance queries. This process builds global dependencies between queries, prompting different queries to differentiate and focus on distinct individual tree targets, thereby suppressing redundant predictions where multiple queries respond to the same target at the feature level. Subsequently, a masked cross-attention mechanism drives the interaction between queries and the voxel features output by the backbone. Distinct from global full-attention mechanisms, this step employs the binary mask predicted in the preceding hierarchy as an attention bias, restricting the queries to aggregate features solely from their corresponding foreground regions. This spatial constraint strategy filters out background noise and interference from neighboring trees, ensuring that query vectors focus on the local fine-grained textures of the target canopy. Finally, a feed-forward network (FFN) performs non-linear transformations on the aggregated features to complete the state update for the current layer.

#### 2.5.2. Spatial Proximity-Weighted Module (SPWM)

Transforming high-dimensional query vectors into precise 3D spatial masks constitutes the final step of individual tree segmentation. Existing methods, such as Organ3DNet, typically rely solely on the dot-product similarity between queries and features to generate masks. However, in forests with high canopy density, the crowns of adjacent trees are interlaced, and the local geometric textures of branches and leaves are highly similar. Consequently, relying exclusively on feature similarity makes it difficult to delineate clear boundaries in overlapping regions. To address this, this study designs the SPWM (Figure 5A), which introduces an explicit geometric center constraint on top of semantic matching.

The core architecture of the SPWM comprises dual-stream prediction paths. In addition to the conventional semantic mask generation branch, we introduce an additional lightweight center regression head. This branch consists of a two-layer MLP designed to map high-dimensional instance queries into 3D physical space. For the *k*-th instance query and the *i*-th point in space, their joint score *S_k_*_,*i*_ is defined as a weighted combination of a semantic consistency term and a geometric penalty term:

(3) $$S_{k,i}=\left(Q_{k}\times F_{i}^{T}\right)-\lambda \times D(P_{i}\times C_{k})$$

where *Q_k_* denotes the *k*-th instance query, *F_i_* represents the feature vector of the *i*-th point, and λ is a balancing coefficient used to control the intensity of the geometric constraint. *D* is a distance penalty function utilized to suppress outliers far from the instance center. To eliminate scale differences across different forest plots, the normalized instance center *C_k_* is first predicted, and its normalized Euclidean distance to the point cloud coordinate *P_i_* is calculated:

(4) $$D\left(P_{i}\times C_{k}\right)={\left\|\frac{P_{i}-P_{min}}{P_{max}-P_{min}}-C_{k}\right\|}_{2}^{2}$$

where *P_min_* and *P_max_* represent the minimum and maximum values of the input point cloud across three dimensions, respectively. $C_{k}\in {\left[0,1\right]}^{3}$ is the geometric centroid predicted from the query vector via the regression branch, constrained by a Sigmoid function to ensure numerical stability. It is worth noting that this geometric constraint exhibits a hierarchical, iterative nature. As the decoder layers deepen, the query vector *Q_k_* gradually incorporates richer contextual information. Consequently, its predicted center *C_k_* progressively approaches the true physical centroid of the tree from an initial random position, thereby enabling the geometric penalty term *D* to generate a more precise spatial attenuation field. This mechanism forces the network to prioritize retaining spatially proximal points while maintaining semantic consistency, effectively severing branch adhesions between adjacent trees and achieving precise separation at the instance level.

### 2.6. Statistical Analysis

To evaluate the segmentation performance of TreeSeg-Net on forest point clouds, this study stratified the assessment process into two dimensions: semantic segmentation and instance segmentation. All metrics were derived from a point-wise comparison between the predicted results and the manually annotated ground truth. In terms of semantic segmentation, *precision*, *recall*, *F*1-score, and *IoU* were employed to assess the model’s accuracy in distinguishing tree foregrounds from background noise. Specifically, the F1-score comprehensively reflects classification robustness, while IoU measures the degree of geometric spatial overlap between the predicted and ground truth point sets. The formulas for these metrics are as follows:

(5) $$Prec=\frac{TP}{TP+FP}$$

(6) $$Rec=\frac{TP}{TP+FN}$$

(7) $$F1=\frac{2\times Prec\times Rec}{Prec+Rec}$$

(8) $$IoU=\frac{TP}{TP+FP+FN}$$

where *TP*, *FP*, and *FN* represent the number of correctly identified, false positive, and false negative tree points, respectively. To evaluate the model’s capability in isolating individual trees from complex forest environments, this study utilized average precision (*AP*), mean precision (*mPrec*), mean recall (*mRec*), mean coverage (*mCov*), and mean weighted coverage (*mWCov*). Average precision serves as the primary metric for instance segmentation. It is defined as the mean AP averaged over IoU thresholds from 0.50 to 0.95. Additionally, the specific metrics AP_50_ and AP_25_ were calculated at IoU thresholds of 0.50 and 0.25, respectively. These metrics first calculate independent scores for each semantic category (e.g., ground, trees) and subsequently take their arithmetic mean to perform a global evaluation. Notably, mCov and mWCov intuitively quantify the extent to which predicted instances restore the true tree crown shapes. The definitions of these metrics are as follows:

(9) $$AP=\int_{0}^{1}Prec\left(Rec\right)dRec$$

(10) $$mPrec=\frac{1}{C}\sum_{i=1}^{C}{Prec}_{i}$$

(11) $$mRec=\frac{1}{C}\sum_{i=1}^{C}{Rec}_{i}$$

(12) $$mCov=\frac{1}{M}\sum_{k=1}^{M}\underset{j}{\max}IoU(G_{k},P_{j})$$

(13) $$mWCov=\sum_{k=1}^{M}\underset{j}{w_{k}\max}IoU(G_{k},P_{j})$$

where *C* is the number of semantic categories, and *Prec_i_* and *Rec_i_* denote the precision and recall of the *i*-th class, respectively. *M* represents the total number of ground truth trees in the plot, while *G_k_* and *P_j_* denote the point sets of the *k*-th ground truth tree and the *j*-th predicted instance, respectively. A detection is considered correct when the IoU between the predicted instance and the ground truth exceeds 0.5. *w_k_* indicates the proportion of points in the *k*-th tree relative to the total number of tree points in the entire plot.

## 3. Results

### 3.1. Platform Configuration and Network Structure

The model proposed in this study was implemented based on the PyTorch 2.2.0 deep learning framework and trained and tested on the Ubuntu 22.04 operating system. The experimental hardware platform consisted of two Intel Xeon Gold 6246R CPUs (@3.40 GHz) and one NVIDIA Quadro RTX 8000 GPU with 48 GB of VRAM, equipped with 128 GB of system memory. The software environment included Python 3.10.9, CUDA 11.8, and cuDNN 8.7.0 to accelerate computations. During the training phase, the AdamW optimizer was employed with an initial learning rate set to 2 × 10^−4^, adjusted using the OneCycleLR policy. The model training was conducted for 300 epochs with a Batch Size of 10. The voxelization size for the input point cloud was set to 0.15. Table 1 details the network configuration of TreeSeg-Net, covering the specific kernel size, stride, and channel dimensions for each stage.

### 3.2. Semantic Segmentation Results

Table 2 presents the semantic segmentation results of TreeSeg-Net on the test set. The results indicate that TreeSeg-Net achieved robust accuracy across both semantic categories, reaching an mIoU of 99.70% and an average F1-score of 99.85%. Specifically, the extraction of vegetation points demonstrated extremely high precision, with the IoU for the tree category reaching 99.55% and a recall rate as high as 99.86%. These metrics suggest that the network effectively separates complex unstructured branch networks from the terrain background, providing high-quality input data for subsequent instance segmentation tasks.

**Figure 6:**
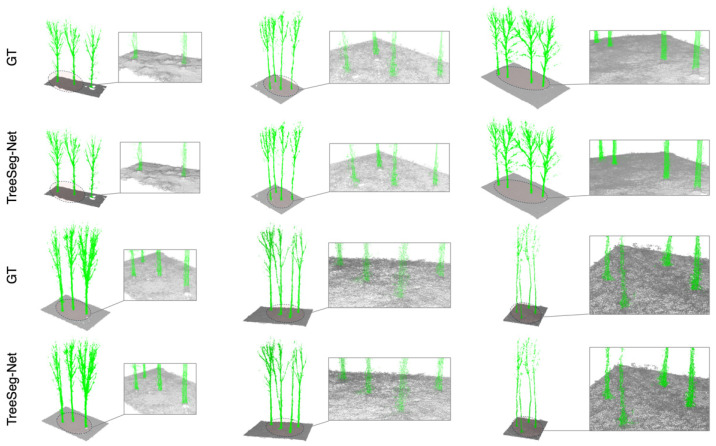
Visual comparison of semantic segmentation results, illustrating the contrast between the GT and the predictions generated by TreeSeg-Net.

Figure 6 illustrates the comparison between the ground truth (GT) and the predicted results. As observed in the figure, manual annotations contain errors at the interface between tree roots and the ground, where parts of the tree trunk bases were incorrectly classified as ground points. This inaccuracy arises from the difficulty of achieving perfectly precise manual delineation on undulating terrain. In contrast, the predictions of TreeSeg-Net corrected these errors, completely preserving the trunk roots within the tree category. This demonstrates that the model learned the morphological features of trees rather than overfitting the errors present in the labels. Consequently, the segmentation details at the tree bases were actually more accurate than the manually annotated GT.

### 3.3. Instance Segmentation Results

Table 3 presents the results of TreeSeg-Net on the instance segmentation task. Compared to the baseline, TreeSeg-Net demonstrated significant performance improvements in the extraction of tree instances. The baseline achieved an AP of 0.825 for the tree category, whereas TreeSeg-Net elevated this metric to 0.972. Regarding coverage metrics, the mWCov of our model reached 0.988, indicating that the predicted instance masks exhibit a high degree of overlap with the real trees in terms of weighted volume. These data reflect that the model can effectively perform individual tree separation when processing unstructured forest scenes.

**Figure 7:**
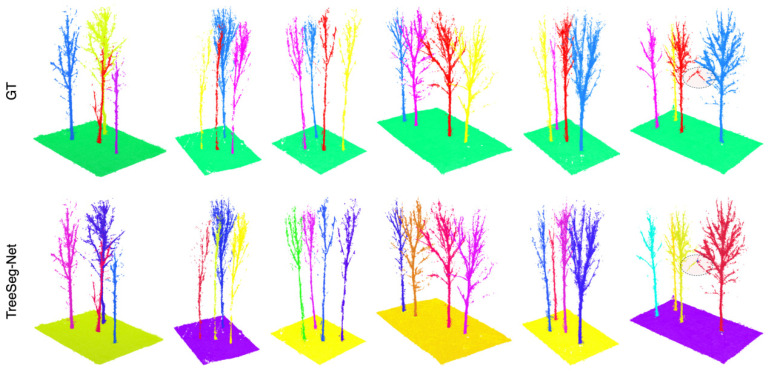
Visualization of instance segmentation results, where different colors represent distinct individual tree instances.

Figure 7 visualizes the instance segmentation results of TreeSeg-Net, where distinct colors denote separate individual tree instances distinguished by the model. From a holistic perspective, TreeSeg-Net accurately delineated closely adjacent trees, exhibiting no significant signs of under-segmentation or over-segmentation. A comparison of the local region on the right side of the figure reveals that the manual GT annotations exhibited fragmentation when processing fine branches, erroneously labeling branches of the same tree as two discontinuous parts. In contrast, leveraging the spatial continuity of the point cloud, TreeSeg-Net correctly merged these fragmented branches into the main trunk instance, thereby preserving the structural integrity of the individual tree. This result suggests that the model, to a certain extent, mitigated the issue of instance discontinuity present in manual annotations. Although the model rectified these subtle annotation errors, the quantitative metrics remained superior due to the exceptionally high overall overlap.

### 3.4. Ablation Studies

To validate the effectiveness of the core modules within TreeSeg-Net and their contributions to the overall model performance, ablation experiments were conducted on the test set. The results are presented in Table 4. This section primarily analyzes the specific performance of GCAM and SPWM in the instance segmentation task.

The baseline model comprises solely the sparse convolutional backbone and a fundamental Transformer decoder. As indicated by the data in the table, the baseline model already exhibited high accuracy in semantic segmentation, achieving an mIoU of 99.50%. This is attributed to the robust feature extraction capabilities of sparse convolution when processing point cloud data. However, in the more challenging instance segmentation task, its AP and precision were 82.50% and 68.50%, respectively, indicating that relying on the basic architecture leaves room for improvement when handling complex forest scenes.

Upon introducing GCAM to the baseline model, all instance segmentation metrics showed varying degrees of improvement. The AP increased from 82.50% to 86.30%, and AP_50_ rose to 87.60%. This result suggests that by integrating long-range contextual dependencies, GCAM enhanced the model’s feature perception capabilities for large-scale forest scenes, thereby strengthening the decoder’s discrimination of instances. Similarly, the independent introduction of SPWM brought significant performance gains. Compared to the baseline, AP increased to 89.30%, with mCov and mWCov reaching 95.10% and 95.70%, respectively. This demonstrates that the geometric center constraint and distance penalty mechanism introduced in SPWM effectively suppressed outlier noise, rendering the predicted instance masks spatially more compact and continuous.

The TreeSeg-Net model, which integrates both GCAM and SPWM, achieved the optimal experimental results. Compared to the baseline model, the AP of TreeSeg-Net rose to 97.20%, instance precision significantly improved from 68.50% to 88.80%, and recall reached 99.20%. This substantial performance boost demonstrates a favorable synergistic effect between GCAM and SPWM in terms of feature enhancement and geometric constraints. Furthermore, it is worth noting that the fluctuations in semantic segmentation metrics across all variants were minimal, indicating that the model maintained robustness in semantic category recognition while enhancing instance segmentation capabilities.

### 3.5. Comparison with Other Networks

To evaluate the efficacy of TreeSeg-Net in complex forest scenes, comparative experiments were conducted on the same dataset against four mainstream point cloud segmentation networks: PointGroup [29], SoftGroup [30], OneFormer3D [39], and Organ3DNet [37]. All comparative models were trained based on the official source code under recommended parameter settings and terminated upon the full convergence of the training loss curves. Table 5 lists the quantitative evaluation results for each model on semantic and instance segmentation tasks.

In the semantic segmentation task, OneFormer3D achieved the highest mIoU of 99.94%, indicating the advantage of Transformer-based architectures in point cloud semantic feature extraction. TreeSeg-Net achieved an mIoU of 99.70%, validating the effectiveness of the sparse convolutional backbone combined with the GCAM module in extracting complex branch features of deciduous forests. In contrast, while PointGroup achieved an instance segmentation AP of 91.87%, its semantic segmentation mIoU was only 87.20%, lower than the method proposed in this study.

The results in Table 5 indicate that TreeSeg-Net outperformed the other four networks across the vast majority of evaluation metrics. Regarding AP, which reflects segmentation quality, TreeSeg-Net reached 97.20%, surpassing PointGroup by 5.33%. This margin highlights the significant advantage of TreeSeg-Net when processing unstructured forest scenes. In comparison, SoftGroup achieved an AP of 82.20%, suggesting that relying solely on semantic features and bottom-up clustering strategies is inadequate for handling complex interlaced branch structures. OneFormer3D achieved the highest recall of 100.00%; however, its AP value was only 86.11%, and its AP_50_ was also lower than that of our method. This phenomenon suggests that while OneFormer3D detected nearly all tree targets, the quality of the generated masks was inferior, prone to redundant predictions or confidence ranking biases. A similar trend appeared with Organ3DNet; despite a recall of 93.10%, its precision was only 68.50%. Conversely, TreeSeg-Net maintained a high level of precision at 88.80% while upholding an extremely high recall, achieving the best value of 97.30% in AP_50_. This is attributed to the SPWM module, which imposes explicit geometric spatial constraints, effectively suppressing under-segmentation and over-segmentation phenomena caused by feature similarity.

## 4. Discussion

High-precision ITS serves as the foundation for extracting phenotypic parameters and estimating carbon stocks from complex forest point clouds. This study investigated the viability of an end-to-end instance segmentation approach for complex forest scenarios by creating a high-density point cloud dataset derived from UAV SfM technology. Although UAV SfM technology provides a low-cost data source, achieving automated and high-precision individual segmentation in high-density deciduous stands remains a formidable challenge [40,41,42]. Existing ITS methods are primarily categorized into traditional clustering-based algorithms and data-driven deep learning algorithms [43,44,45]. While traditional region growing or watershed algorithms perform well in sparse stands, they often exhibit limitations such as over-segmentation or under-segmentation when dealing with complex overlapping crowns due to an over-reliance on local geometric features [46].

In contrast, deep learning methods demonstrate robust feature extraction capabilities. The fundamental innovation of TreeSeg-Net lies in the incorporation of GCAM and SPWM within a sparse 3D U-Net architecture. Specifically, SPWM amplifies the feature disparity between the crown center and its edge regions by incorporating spatial distance information [47]. This mechanism effectively prevents adjacent trees with highly similar branch textures from being erroneously merged, which notably improves the model’s performance in scenarios characterized by severe canopy interlacing. Simultaneously, GCAM establishes long-range dependencies between feature points via attention mechanisms, compensating for the limited local receptive field of the sparse convolutional backbone [48,49]. By addressing point cloud discontinuity caused by sparse leaves or mutual occlusion, this module integrates spatially discrete local features into a holistic entity, ensuring the geometric structural integrity and continuity of tree instances.

When compared to mainstream clustering-based networks such as PointGroup and SoftGroup, our approach demonstrates substantial improvements in boundary delineation and instance separation. This advantage primarily stems from the end-to-end mask prediction paradigm adopted by TreeSeg-Net [50,51]. Existing methods often suffer from under-segmentation in dense stands because they rely on bottom-up clustering, which struggles when the semantic features of adjacent branches are indistinguishable [21,52]. In contrast, our SPWM imposes a distance penalty that forces the network to prioritize spatially proximal points, thereby establishing clear segmentation boundaries even in overlapping regions.

However, it is acknowledged that point clouds reconstructed via SfM algorithms are inherently limited by lighting conditions and shooting angles. As observed in our study, this often results in missing trunk information in the lower canopy compared to LiDAR data, which restricts the direct application of the model for diameter at breast height extraction. In contrast, airborne or terrestrial LiDAR can provide more complete vertical structures but comes with high costs and limited portability [53]. Furthermore, the current model training relies primarily on a single type of forest data. The generalization capability of the model when facing mountainous scenes with more complex species compositions or dramatic terrain undulations remains to be further validated through transfer learning or domain adaptation techniques [54,55].

## 5. Conclusions

To address the challenges of ITS in complex forest environments, this paper proposes the TreeSeg-Net instance segmentation model. Built upon a sparse 3D U-Net backbone, the model integrates targeted improvement modules to adapt to the unstructured features of deciduous forest point clouds. Validating the research hypotheses posited in this study, the integration of GCAM effectively compensates for the limited receptive field of sparse convolution to enhance whole-tree structural perception, while the SPWM confirms that explicit geometric spatial constraints are essential for resolving the boundary adhesion issue caused by similar physical features of adjacent crowns in high-density stands. Experimental results demonstrate that TreeSeg-Net outperforms current mainstream individual tree segmentation methods. It achieved an AP of 97.2% and an mWCov of 98.8%. Compared to mainstream deep learning methods such as PointGroup and OneFormer3D, TreeSeg-Net exhibits higher precision and robustness in handling canopy overlap, identifying fine boundaries, and minimizing over-segmentation and under-segmentation errors. These findings substantiate the critical roles of global context enhancement and geometric spatial constraint strategies in improving segmentation performance. In summary, the end-to-end network constructed in this study realizes the precise extraction of individual tree instances from forest point clouds without manual intervention, providing strong technical support for efficient forest resource inventory and refined management.

## Figures and Tables

**Table 1:** Detailed architecture configuration of TreeSeg-Net.

Stage	Module/Component	Operation	Kernel Size	Stride	Out Channels
Stem	Input Conv	MinkConv	5 × 5 × 5	1	32
	Downsample	MinkConv	2 × 2 × 2	2	32
Encoder	Stage 1	ResBlock × 2	3 × 3 × 3	1	32
	Downsample	MinkConv	2 × 2 × 2	2	64
	Stage 2	ResBlock × 3	3 × 3 × 3	1	64
	Downsample	MinkConv	2 × 2 × 2	2	128
	Stage 3	ResBlock × 4	3 × 3 × 3	1	128
	Feature Refine	GCAM	Global Pool	-	128
	Downsample	MinkConv	2 × 2 × 2	2	256
	Stage 4	ResBlock × 6	3 × 3 × 3	1	256
	Feature Refine	GCAM	Global Pool	-	256
Decoder	Upsample 4	MinkTranspose	2 × 2 × 2	2	256
	Stage 5	ResBlock × 2	3 × 3 × 3	1	256
	Upsample 5	MinkTranspose	2 × 2 × 2	2	128
	Stage 6	ResBlock × 2	3 × 3 × 3	1	128
	Upsample 6	MinkTranspose	2 × 2 × 2	2	96
	Stage 7	ResBlock × 2	3 × 3 × 3	1	96
	Upsample 7	MinkTranspose	2 × 2 × 2	2	96
	Stage 8	ResBlock × 2	3 × 3 × 3	1	96
	Feature Refine	GCAM	Global Pool	-	96
Transformer	Query Refine	QRM	-	-	128
Head	Instance Branch (Mask)	MLP	-	-	128 (Embed)
	Instance Branch (Geometric)	MLP	-	-	3 (x, y, z)
	Semantic Branch	MLP	-	-	3 (Class) *

* The output channel is set to 3 to include the ‘no-object’ class required by the Transformer architecture.

**Table 2:** Quantitative comparison of semantic segmentation performance between the baseline and TreeSeg-Net.

Method	Class	Precision (%)	Recall (%)	F1-Score (%)	IoU (%)
Baseline	Ground	99.95	99.87	99.91	99.82
	Tree	99.64	99.86	99.75	99.50
	Average	99.80	99.87	99.83	99.66
TreeSeg-Net	Ground	99.95	99.89	99.92	99.84
	Tree	99.70	99.86	99.78	99.55
	Average	99.83	99.87	99.85	99.70

**Table 3:** Performance evaluation of instance segmentation between the baseline and TreeSeg-Net.

Method	Class	AP	AP_50_	AP_25_	mCov	mWCov
Baseline	Ground	0.988	0.988	0.988	0.998	0.998
	Tree	0.825	0.842	0.855	0.895	0.908
	Average	0.907	0.915	0.922	0.947	0.953
TreeSeg-Net	Ground	0.999	0.999	0.999	0.998	0.998
	Tree	0.972	0.973	0.973	0.981	0.988
	Average	0.986	0.986	0.986	0.990	0.993

**Table 4:** Ablation study of TreeSeg-Net.

Method	Semantic Segmentation (%)				Instance Segmentation (%)					
	Prec	Rec	F1	IoU	AP	AP_50_	Prec	Rec	mCov	mWCov
Baseline	99.64	99.86	99.75	99.50	82.50	84.20	68.50	93.10	89.50	90.80
+GCAM	99.57	99.88	99.73	99.46	86.30	87.60	70.50	94.80	92.20	92.80
+SPWM	99.55	99.89	99.72	99.44	89.30	90.60	72.00	96.70	95.10	95.70
TreeSeg-Net	99.70	99.86	99.78	99.55	97.20	97.30	88.80	99.20	98.10	98.80

**Table 5:** Comparison with other mainstream methods.

Method	mIoU (%)	AP (%)	AP_50_ (%)	AP_25_ (%)	Rec (%)	Prec (%)
PointGroup	87.20	91.87	-	-	-	-
SoftGroup	98.80	82.20	94.40	90.10	91.20	-
OneFormer3D	99.94	86.11	94.64	93.49	100.00	89.47
Organ3DNet	99.66	82.50	84.20	85.50	93.10	68.50
TreeSeg-Net	99.70	97.20	97.30	97.30	99.20	88.80

## Data Availability

Data and code are available upon request.
